# XBB.1.5 mRNA COVID-19 vaccine protection against inpatient or emergency department visits among adults infected with SARS-CoV-2 JN.1 and XBB-lineage variants

**DOI:** 10.3389/fimmu.2025.1470609

**Published:** 2025-02-17

**Authors:** Matthew E. Levy, Vanessa Chilunda, Phillip R. Heaton, Deran McKeen, Jason D. Goldman, Richard E. Davis, Cynthia A. Schandl, William B. Glen, Lisa M. McEwen, Elizabeth T. Cirulli, Dana Wyman, Andrew Dei Rossi, Hang Dai, Magnus Isaksson, Nicole L. Washington, Tracy Basler, Kevin Tsan, Jason Nguyen, Jimmy Ramirez, Efren Sandoval, William Lee, James Lu, Shishi Luo

**Affiliations:** ^1^ Helix, San Mateo, CA, United States; ^2^ Department of Pathology and Laboratory Medicine, HealthPartners, Bloomington, MN, United States; ^3^ HealthPartners Institute, Bloomington, MN, United States; ^4^ Swedish Center for Research and Innovation, Providence Swedish Medical Center, Seattle, WA, United States; ^5^ Division of Allergy and Infectious Disease, University of Washington, Seattle, WA, United States; ^6^ Providence Sacred Heart Medical Center and Children’s Hospital, Spokane, WA, United States; ^7^ Department of Pathology and Laboratory Medicine, Medical University of South Carolina, Charleston, SC, United States

**Keywords:** COVID-19 vaccines, SARS-COV-2 variants, hospitalization, emergency room visits, epidemiology

## Abstract

As part of a multi-state viral genomic surveillance program, we conducted a case-only analysis to evaluate the effectiveness of XBB.1.5-adapated mRNA vaccines in preventing severe illness among individuals with medically attended SARS-CoV-2 infection. We compared prior receipt of an XBB.1.5-adapted mRNA vaccine between SARS-CoV-2-infected adults with inpatient or emergency department (ED) visits (as a proxy for severe illness) vs those with outpatient visits (as a proxy for mild illness). Among 6,551 patients between September 2023 and January 2024, 6.1% with inpatient or ED visits vs 12.0% with outpatient visits had received XBB.1.5 vaccination (adjusted odds ratio [aOR]=0.41; 95% confidence interval [CI]: 0.32-0.53). This protective association was weaker among JN.1 (aOR=0.62; 95% CI: 0.40-0.96) vs XBB-lineage (aOR=0.28; 95% CI: 0.18-0.43) variant infections (interaction, p=0.003). XBB.1.5 vaccines protect against severe illness, but protection may be weaker against JN.1 vs XBB-lineage variants. This study highlights the need for COVID-19 vaccines to be routinely updated to align with circulating strains and for individuals to stay up to date with recommended vaccines.

## 1 Introduction

On 11 September 2023, the US Food and Drug Administration (FDA) approved the 2023-2024 BNT162b2 (Pfizer-BioNTech) and mRNA-1273 (Moderna) monovalent mRNA COVID-19 vaccines for individuals aged ≥12 years, with emergency use authorization granted for children aged 6 months to 11 years. These vaccines target the spike protein of the SARS-CoV-2 Omicron variant XBB.1.5, which was predominant in the US from January to May 2023 (1). The US Centers for Disease Control’s (CDC’s) Advisory Committee on Immunization Practices (ACIP) subsequently recommended that individuals aged ≥6 months receive an XBB.1.5-adapted vaccine regardless of their vaccination history, to enhance protection against circulating variants (2).

By September 2023, other XBB variants such as EG.5 and HV.1 (both sublineages of XBB.1.9.2) had surpassed XBB.1.5 in prevalence. By late December 2023, another novel variant, JN.1 (a sublineage of BA.2.86), had become predominant, accounting for 65% of SARS-CoV-2 infections nationwide by 6 January 2024 (1). The rapid rise of JN.1, which possesses more than 30 mutations in the spike protein compared to XBB.1.5 (including the notable L455S mutation), could be attributed to increased immune escape and infectivity (3, 4). In laboratory-based neutralization studies, JN.1 has displayed increased resistance to neutralization by antibodies induced by XBB.1.5-adapted mRNA vaccination (4–6). However, immunogenicity studies suggest that antibody titers are likely to remain effective (7). Initial findings indicate short-term effectiveness of XBB.1.5-adapted mRNA vaccines for protecting against symptomatic SARS-CoV-2 infection and severe COVID-19 outcomes, largely during periods of XBB predominance (8–14).

Case-only studies among SARS-CoV-2-positive individuals have previously been employed to compare the protection offered by vaccines against different variants (13, 15–17). However, variant-specific estimates of XBB.1.5 vaccine protection against severe COVID-19 outcomes, especially those caused by the JN.1 variant, remain limited. In this study, we leverage a multi-state viral genomic surveillance program to conduct a case-only analysis evaluating the association between XBB.1.5-adapted mRNA vaccination and the likelihood of patients requiring inpatient or emergency department (ED) visits (considered severe) vs outpatient visits (considered mild). This association serves as an indicator of the vaccine’s level of protection against severe illness following infection with a particular variant. We have evaluated this association both overall and separately among patients infected with the JN.1 variant and XBB-lineage variants, enabling a direct comparison of protection against these two distinct SARS-CoV-2 lineages.

## 2 Materials and methods

### 2.1 Design and setting

Within a pan-respiratory virus genomic surveillance program, residual clinical samples from patients who tested positive for a respiratory virus (molecular or antigen) were obtained from three health systems spanning five U.S. states (**Supplementary Table S1**) (18). Samples were initially collected from patients during medical visits and were characterized based on location of collection as inpatient, ED, or outpatient. Patients’ demographic characteristics and COVID-19 vaccination history were extracted from electronic health records (EHRs) and state vaccine registries. Study protocols were reviewed and approved by WIRB CG IRB (Western Institutional Review Board, WIRB-Copernicus Group; approval number 20224919) and the Medical University of South Carolina (MUSC) Institutional Review Board for Human Research (approval number Pro00129083). This study presented minimal risk to participants because there was no interaction or intervention with patients; therefore, the requirement for informed consent was waived.

### 2.2 Viral sequencing

Viral sequencing was performed by Helix using a hybridization-capture based assay (Twist Biosciences) and short-read genome sequencing technology (Illumina), as previously described (19). SARS-CoV-2 was identified in samples with reads that aligned to the reference genome, and lineages were assigned using pangolin version 4.3.1 (**Supplementary Methods**; **Supplementary Figure S1**).

### 2.3 Study sample

This analysis included SARS-CoV-2-positive samples collected from adults aged ≥18 years between 24 September 2023 and 21 January 2024. SARS-CoV-2 infection was identified from either clinical diagnostic testing (performed/ordered by the health system), viral sequencing (performed by Helix), or both.

### 2.4 Visit type

The clinical visit type associated with sample collection was a surrogate measure of the severity of illness at time of testing. Inpatient and ED visits represented more severe illness compared to outpatient visits. The reasons for visits and patients’ specific symptoms were not available for analysis.

### 2.5 Vaccination status

COVID-19 vaccination status was assigned using the date and type of the most recent dose received prior to the specimen collection date. Patients were considered to be: 1) vaccinated with an XBB.1.5-adapted monovalent mRNA vaccine if their last dose occurred on/after September 12, 2023 and was BNT162b2 or mRNA-1273; 2) vaccinated with a BA.4/BA.5-adapted bivalent mRNA vaccine if their last dose occurred between September 1, 2022 and September 11, 2023 and was BNT162b2 or mRNA-1273; 3) vaccinated with an original wild-type monovalent mRNA or viral vector vaccine if their last dose occurred before September 1, 2022 and was either BNT162b2, mRNA-1273, or Ad26.COV2.S; and 4) unvaccinated if they had received no prior COVID-19 vaccine doses. Patients were excluded if they received any dose 0-6 days before the collection date (n=83) or if their most recent dose was a different (n=20) or unknown (n=124) vaccine type.

### 2.6 Statistical analysis

In this case-only analysis among adults with medically attended SARS-CoV-2 infection, the odds of prior XBB.1.5 vaccination were compared between inpatient or ED patients and outpatients. We also compared inpatient vs outpatient (excluding ED). Adjusted odds ratios (aORs) and 95% confidence intervals (CIs) for the association between vaccination status and visit type were calculated using multivariable logistic regression, adjusting for age group, sex, race/ethnicity, health system and state of residence, and collection date (natural cubic spline). In multivariable models, effect modification by variant (JN.1 vs XBB-lineage) was assessed using an interaction term with vaccination status. Receipt of an XBB.1.5 vaccine was compared to no receipt of an XBB.1.5 vaccine (irrespective of vaccination history) and to three specific reference groups: 1) BA.4/BA.5 vaccination but no XBB.1.5 vaccine; 2) wild-type vaccination but no BA.4/BA.5 or XBB.1.5 vaccine; and 3) unvaccinated. In a separate analysis, XBB.1.5 vaccine recipients were further categorized based on duration of time since their dose (7-59 vs ≥60 days earlier). In addition, BA.4/BA.5 and wild-type vaccine recipients were compared to unvaccinated. Subgroup analyses were conducted among patients infected with JN.1, XBB-lineage (any), HV.1, and EG.5 variants; among patients aged ≥65 years; and among patients with no other respiratory virus coinfection. *P*<0.05 was considered statistically significant and analyses were performed using R version 4.2.3.

## 3 Results

Among 6,551 adults with medically attended SARS-CoV-2 infection, 1,912 (29.2%) were tested in either an inpatient (1,012; 15.4%) or ED (900; 13.7%) setting, while 4,639 (70.8%) were tested in an outpatient setting. Most SARS-CoV-2 infections were detected through clinical diagnostic testing, with only 8 first identified through viral sequencing. Patient characteristics stratified by visit type are presented in **Table 1**. Inpatients had a higher median age (73 years; IQR: 61-82) compared to ED patients (55 years; IQR: 35-72) and outpatients (52 years; IQR: 36-68). Lineages were successfully assigned to 4,480 samples (68.4%), with the most prevalent variants being JN.1 (1,084; 24.2%), HV.1 (803; 17.9%), and EG.5 (746; 16.7%). During the most recent 2-week period ending 20 January 2024, JN.1 accounted for 73.1% of sequenced samples, while HV.1 (5.5%) and EG.5 (2.9%) were less prevalent, consistent with national data (1).

**Table 1:** Patient characteristics overall and stratified by visit type.

Characteristic	Overall (n=6,551), No. (%)	Visit type		
		Inpatient (n=1,012), No. (%)	ED (n=900), No. (%)	Outpatient (n=4,639), No. (%)
Health system				
HealthPartners	5,466 (83.4)	448 (44.3)	566 (62.9)	4,452 (96.0)
Medical University of South Carolina	202 (3.1)	113 (11.2)	61 (6.8)	28 (0.6)
Providence Health and Services	883 (13.5)	451 (44.6)	273 (30.3)	159 (3.4)
Month of specimen collection				
September 2023	410 (6.3)	62 (6.1)	62 (6.9)	286 (6.2)
October 2023	1,385 (21.1)	251 (24.8)	185 (20.6)	949 (20.5)
November 2023	1,604 (24.5)	220 (21.7)	223 (24.8)	1,161 (25.0)
December 2023	2,316 (35.4)	316 (31.2)	307 (34.1)	1,693 (36.5)
January 2024	836 (12.8)	163 (16.1)	123 (13.7)	550 (11.9)
Age group				
18 to 49 y	2,684 (41.0)	138 (13.6)	387 (43.0)	2,159 (46.5)
50 to 64 y	1,428 (21.8)	163 (16.1)	176 (19.6)	1,089 (23.5)
65 to 74 y	1,106 (16.9)	246 (24.3)	137 (15.2)	723 (15.6)
75 to 84 y	904 (13.8)	270 (26.7)	135 (15.0)	499 (10.8)
≥85 y	429 (6.5)	195 (19.3)	65 (7.2)	169 (3.6)
Female	3,893 (59.4)	505 (49.9)	523 (58.1)	2,865 (61.8)
Race and ethnicity				
Asian, non-Hispanic	325 (5.0)	23 (2.3)	20 (2.2)	282 (6.1)
Black, non-Hispanic	828 (12.6)	88 (8.7)	146 (16.2)	594 (12.8)
Hispanic	256 (3.9)	18 (1.8)	41 (4.6)	197 (4.2)
White, non-Hispanic	3,851 (58.8)	432 (42.7)	413 (45.9)	3,006 (64.8)
Other or unknown	1,291 (19.7)	451 (44.6)	280 (31.1)	560 (12.1)
COVID-19 vaccination statusa				
Unvaccinated	1,842 (28.1)	360 (35.6)	358 (39.8)	1,124 (24.2)
Vaccinated with a wild-type vaccine	2,879 (43.9)	397 (39.2)	386 (42.9)	2,096 (45.2)
Vaccinated with a BA.4/BA.5 vaccine	1,155 (17.6)	182 (18.0)	112 (12.4)	861 (18.6)
Vaccinated with an XBB.1.5 vaccine	675 (10.3)	73 (7.2)	44 (4.9)	558 (12.0)
SARS-CoV-2 variant^b^				
JN.1	1,084 (16.5)	139 (13.7)	165 (18.3)	780 (16.8)
HV.1	803 (12.3)	116 (11.5)	114 (12.7)	573 (12.4)
EG.5	746 (11.4)	119 (11.8)	119 (13.2)	508 (11.0)
XBB.1.16.1	379 (5.8)	54 (5.3)	67 (7.4)	258 (5.6)
XBB.1.5	262 (4.0)	44 (4.3)	47 (5.2)	171 (3.7)
HK.3	231 (3.5)	39 (3.9)	33 (3.7)	159 (3.4)
XBB.1.16.6	219 (3.3)	34 (3.4)	43 (4.8)	142 (3.1)
XBB.2.3	209 (3.2)	31 (3.1)	24 (2.7)	154 (3.3)
FL.1.5.1	151 (2.3)	24 (2.4)	26 (2.9)	101 (2.2)
BA.2.86	146 (2.2)	20 (2.0)	23 (2.6)	103 (2.2)
XBB.1.9.1	38 (0.6)	6 (0.6)	5 (0.6)	27 (0.6)
XBB.1.16	37 (0.6)	5 (0.5)	7 (0.8)	25 (0.5)
XBB.1.9.2	20 (0.3)	7 (0.7)	3 (0.3)	10 (0.2)
Other XBB	98 (1.5)	15 (1.5)	9 (1.0)	74 (1.6)
Other non-XBB	57 (0.9)	6 (0.6)	9 (1.0)	42 (0.9)
Insufficient sequencing data	2,071 (31.6)	353 (34.9)	206 (22.9)	1,512 (32.6)

ED, emergency department.

a Defined by whether there was ≥1 COVID-19 vaccine record prior to the specimen collection date as well as by the date and type of the most recent dose received.

b SARS-CoV-2 variant lineages were assigned using pangolin version 4.3.1. Except for HV.1 and HK.3, sublineages of EG.5 are aggregated with EG.5. Except for JN.1, sublineages of BA.2.86 are aggregated with BA.2.86. Except for FL.1.5.1, sublineages of XBB.1.9.1 are aggregated with XBB.1.9.1. Except for XBB.1.16.1 and XBB.1.16.6, sublineages of XBB.1.16 are aggregated with XBB.1.16. Except for EG.5, HV.1, and HK.3, sublineages of XBB.1.9.2 are aggregated with XBB.1.9.2. Sublineages of each other named lineage are aggregated with the respective lineage. JN.1 is also known as BA.2.86.1.1; HV.1 as XBB.1.9.2.5.1.6.1; EG.5 as XBB.1.9.2.5; HK.3 as XBB.1.9.2.5.1.1.3; and FL.1.5.1 as XBB.1.9.1.1.5.1.

Regarding vaccination status, 675 (10.3%) patients had received XBB.1.5 vaccination (a median of 57 days earlier [IQR: 39-73; range: 7-122]), 1,155 (17.6%) had received BA.4/BA.5 vaccination (but not XBB.1.5 vaccination) (median of 374 days since last dose [IQR: 330-414]), 2,879 (43.9%) had received wild-type vaccination (but not BA.4/BA.5 or XBB.1.5 vaccination) (median of 712 days since last dose [IQR: 625-818]), and 1,842 (28.1%) were unvaccinated. Among XBB.1.5-vaccinated patients, the median time since vaccination was 64 days for JN.1 infections (IQR: 51-80) and 52 days for XBB-lineage infections (IQR: 31-64). In the most recent 14-day period, 19.4% of patients overall were XBB.1.5-vaccinated.

**Figure 1:**
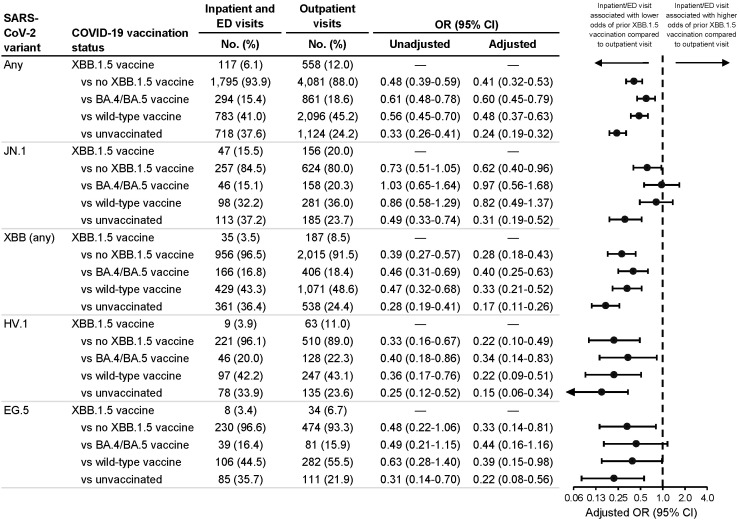
Association between inpatient or emergency department vs outpatient visit type and prior receipt of an XBB.1.5 vaccine. Associations were calculated among all SARS-CoV-2 infections and among JN.1, XBB (any sublineage), HV.1, and EG.5 infections. Odds ratios (ORs) were calculated comparing prior receipt of an XBB.1.5 vaccine to no prior receipt of an XBB.1.5 vaccine (irrespective of previous COVID-19 vaccination history) as well as to each of three specific reference groups: 1) prior receipt of a BA.4/BA.5 vaccine but not an XBB.1.5 vaccine; 2) prior receipt of a wild-type vaccine but not a BA.4/BA.5 or XBB.1.5 vaccine; and 3) unvaccinated. Adjusted ORs were adjusted for age group (18-49, 50-64, 65-74, 75-84, and ≥85 years), sex, race and ethnicity (Asian, non-Hispanic; Black, non-Hispanic; Hispanic; white, non-Hispanic; and other/unknown), health system and state of residence, and collection date (natural cubic spline with 4 degrees of freedom). CI indicates confidence interval; ED, emergency department.

Among all SARS-CoV-2 infections, 6.1% of patients with inpatient/ED visits had received XBB.1.5 vaccination, compared to 12.0% of patients with outpatient visits (**Figure 1**). In multivariable analysis, XBB.1.5 vaccination a median of 57 days earlier vs no XBB.1.5 vaccination was associated with lower odds of inpatient/ED visits compared to outpatient visits (aOR=0.41; 95% CI: 0.32-0.53). This protective association was significant among any-variant infections regardless of the specific reference group used: vs BA.4/BA.5 vaccination a median of 374 days earlier (aOR=0.60; 95% CI: 0.45-0.79); vs wild-type vaccination a median of 712 days earlier (aOR=0.48; 95% CI: 0.37-0.63); and vs unvaccinated (aOR=0.24; 95% CI: 0.19-0.32).

When stratifying by variant, patients with XBB.1.5 vaccination (vs. no XBB.1.5 vaccination) had lower odds of inpatient/ED visits among JN.1 infections (aOR=0.62; 95% CI: 0.40-0.96) and among XBB-lineage infections (aOR=0.28; 95% CI: 0.18-0.43) (**Figure 1**), but this association was weaker among JN.1 vs XBB-lineage infections (vaccination-variant interaction, p=0.003). This interaction between vaccination and JN.1 vs XBB-lineage variant remained significant when comparing XBB.1.5-vaccinated patients to BA.4/BA.5-vaccinated (p=0.009), wild-type-vaccinated (p=0.003), and unvaccinated (p=0.035) patients. Regarding specific vaccination reference groups, protective associations for XBB.1.5 vaccination were strongest when comparing XBB.1.5-vaccinated to unvaccinated patients.

In additional analyses, findings were similar when only including adults aged ≥65 years (**Supplementary Figure S2**; vaccination-variant interaction, p=0.043), when comparing only inpatient vs outpatient visits (**Supplementary Figure S3**; interaction, p=0.013), and when excluding patients with respiratory virus coinfections (**Supplementary Figure S4**; interaction, p=0.005). Among JN.1 and XBB-lineage infections, similar protective associations were detected for patients XBB.1.5-vaccinated 7-59 days earlier and ≥60 days earlier (**Supplementary Figure S5**). BA.4/BA.5 vaccination and wild-type vaccination were also associated with lower odds of inpatient/ED visits compared to unvaccinated (**Supplementary Figure S6**).

## 4 Discussion

In this multi-state study of adults with medically attended SARS-CoV-2 infection between September 2023 and January 2024, XBB.1.5 mRNA-vaccinated individuals had an overall 59% lower odds of having an inpatient or ED visit vs outpatient visit. Unlike numerous other observational studies examining XBB.1.5 vaccination and severe COVID-19 outcomes (8, 9, 11, 14), our study provides novel data on XBB.1.5 vaccine-associated protection against specific contemporary circulating variants. This was made possible through the linkage of viral sequencing and patient-level clinical data, which is a key component of genomic surveillance programs that enhances their capacity to evaluate vaccine effectiveness against specific SARS-CoV-2 variants (20).

While findings provide evidence that XBB.1.5 vaccines protected against severe illness associated with both JN.1 and XBB-lineage variants, protection against JN.1 was significantly lower. Among JN.1-infected patients, XBB.1.5 vaccination was associated with 38% lower odds of inpatient/ED vs outpatient visits, compared to the 72% lower odds observed among XBB-lineage-infected patients. Other studies have similarly reported lower estimates for vaccine effectiveness against JN lineages compared to XBB lineages, even when comparing similar intervals since vaccination (10, 13, 21, 22). JN.1 has more than 30 spike protein mutations compared to the XBB.1.5 spike protein, including the L455S mutation which is hypothesized to contribute to increased immune evasion (3). In contrast, XBB lineages such as EG.5 and HV.1 are more genetically similar to XBB.1.5, which could explain why XBB.1.5-adapted vaccines may be less effective against the JN.1 variant than against XBB-lineage variants. This key finding emphasizes the need for COVID-19 vaccines to be routinely updated to align with circulating strains and for individuals to stay up to date with recommended vaccines. This may be particularly evident when saltational evolution occurs, as happened to produce BA.2.86. With the addition of L455S, the JN.1 variant has displaced XBB variants.

Given that associations among JN.1-infected individuals were similar irrespective of time since XBB.1.5 vaccination, differences in protection by variant were not attributed to waning effectiveness over the approximately four months of available data. Within the sample of JN.1-infected individuals, this finding was based on a comparison of receipt of vaccination either 7-59 days earlier or 60-122 days earlier, which could have masked differences within or beyond the time frames examined. In other studies of XBB.1.5 vaccine effectiveness, there has been mixed evidence on waning effectiveness, likely related to the durations of follow-up and/or whether studies classified infections based on variant lineage. Several short-term studies found little evidence of waning effectiveness through 3-5 months post-vaccination (10, 22–26). Other studies found that effectiveness waned over time, yet individual-level sequencing data were often unavailable, and temporal differences in variant circulation may have confounded results (21, 27, 28). These findings underscore the importance of continued genomic surveillance and longer-term follow-up to better understand the durability of protection and to inform future vaccine formulation strategies.

The inverse association between XBB.1.5 vaccination (administered a median of 57 days earlier) and inpatient/ED visit type was consistently observed regardless of the specific reference group used, including prior receipt of BA.4/BA.5 vaccination approximately one year earlier, on average, without subsequent XBB.1.5 vaccination. Thus, the protection associated with XBB.1.5 vaccination was enhanced beyond the remaining immunity conferred by prior BA.4/BA.5 vaccination administered during the previous respiratory illness season. However, because vaccination subgroups varied in both vaccine type and time since last vaccination, we cannot conclusively determine whether the observed protection is primarily attributable to the vaccine type or to declining neutralizing antibody titers over time. It is also important to note that results were obtained within the context of widespread natural immunity from prior infection, which could have resulted in attenuated associations if individuals who had not received XBB.1.5 vaccination were more likely to have infection-induced immunity. Thus, estimated vaccine protection represents the incremental benefit of XBB.1.5 vaccination in a population with high levels of infection-induced and/or vaccine-induced immunity.

In this case-only study of SARS-CoV-2-positive patients with sequenced samples, we compared the vaccination status of inpatients or ED patients with that of outpatients. This measure of association provides an indicator of the vaccine’s level of protection against severe vs mild illness and facilitates a comparison across circulating variants. Other studies of XBB.1.5-adapated vaccines, either cohort studies or test-negative case-control studies, have evaluated protection against both severe and mild illness (although not for specific variants), and had similar conclusions regarding the relative protection against severe vs mild illness. In one study, effectiveness was 60% against hospitalization compared to 33% against any medically attended COVID-19, representing 82% greater protection against severe illness (29). In another study, effectiveness was 77% against hospitalization compared to 65% against symptomatic infection, representing 16% greater protection against severe illness (30). Similar patterns were also reported in studies of earlier COVID-19 vaccines. In a study of BA.4/BA.5-adapted bivalent mRNA vaccination, effectiveness was 61% against hospitalization, 46% against ED or urgent care visits, and 35% against outpatient visits (31). In a study of the original wild-type vaccines, the odds that hospitalized patients had moderate or severe illness (vs ‘mild’ illness, i.e., hospitalized for ≤24 hours) was 63%-68% lower in vaccinated vs unvaccinated individuals (32).

This study has several limitations. First, although all patients had laboratory-confirmed SARS-CoV-2 infection at a medical visit, we lacked data on symptoms and reasons for visits. Second, visit type was assessed upon sample collection for SARS-CoV-2 testing and it is unknown whether outpatients later had ED or inpatient visits, potentially contributing to misclassification. Third, we lacked data on social factors (e.g., insurance type) and underlying medical conditions, which could have resulted in residual confounding. Fourth, misclassification of vaccination status is possible if vaccine doses documented in EHRs and registries were incomplete. Vaccination status was defined based on the most recent vaccine dose administered and XBB.1.5-vaccinated individuals were not able to be further classified based on the receipt and timing of earlier booster doses. Taken together, these limitations may have introduced biases but are unlikely to account for observed differences by variant, particularly given that the infecting variant was not yet known at the time of medically attended testing. Finally, data availability for the current study only allowed for an evaluation of vaccine protection through a maximum interval of approximately four months post-vaccination; thus, we were unable to analyze durability over a longer time frame.

This study provides evidence of the effectiveness of XBB.1.5-adapted mRNA vaccines in protecting against severe illness requiring inpatient or ED visits among adults infected with JN.1 or XBB-lineage variants. These results support the prior recommendation that all adults should, irrespective of their previous COVID-19 vaccination history, receive the 2023-2024 COVID-19 vaccine to enhance their protection. However, findings also suggest that XBB.1.5 vaccines provide comparatively less protection against the JN.1 variant than they do against earlier XBB-lineage variants. Future research should continue to confirm the degree of COVID-19 vaccine protection against severe illness associated with infection with emerging SARS-CoV-2 variants and to accordingly evaluate the potential for waning effectiveness over longer durations of follow-up post-vaccination.

## Data Availability

The datasets presented in this article are not readily available because data sharing agreements between Helix and partner institutions prohibit Helix from making this dataset publicly available. Requests to access the datasets should be directed to ML (matt.levy@helix.com) and SL (shishi.luo@helix.com).
