# Supplementary material for: XBB.1.5 mRNA COVID-19 vaccine protection against inpatient or emergency department visits among adults infected with SARS-CoV-2 JN.1 and XBB-lineage variants
Source: Front Immunol. 2025 Feb 17;16:1470609. doi: 10.3389/fimmu.2025.1470609 (PMC11872700; doi:10.3389/fimmu.2025.1470609)
Supplement: Supplementary file 1 [file DataSheet1.pdf]

Supplementary Methods:

Viral sequencing, bioinformatic analyses, and SARS-CoV-2 lineage designation

Viral sequencing

RNA was extracted from 400 µL of remnant patient anterior nares samples using the MagMAX Viral/Pathogen kit (ThermoScientific). RNA libraries (5µL RNA input volume) were generated using the Rapid RNA Library Kit protocol (Swift Biosciences/Integrated DNA Technologies). SARS-CoV-2 genome was enriched and captured using the Respiratory Virus Research Panel hybridization probe panel (Twist Biosciences). Samples were sequenced using the NovaSeq 6000 Sequencing system S1 flow cell, with S1 Reagent Kit v1.5 (300 cycles).

Bioinformatic analyses

The flow cell output was demultiplexed with bcl2fastq (Illumina) into per-sample FASTQ sequences. These sequences were then processed using the Helix fastagenerator pipeline to produce a consensus sequence FASTA file. First, reads were aligned to a reference set consisting of a representative genome of each respiratory virus that was targeted by the hybridization probes, and the human transcriptome (GENCODE v37) using BWA-MEM. The non-human reads were then re-aligned to an expanded set of respiratory viruses reference genomes including the SARS-CoV-2 genome (NCBI accession NC_045512.2) using a de Bruijn graph based algorithm (based on https://doi.org/10.1093/bioinformatics/btz814) to identify sequences that best match the reads. SARS-CoV-2 aligned reads were then marked for duplicates followed by variant

calling using the Haplotyper algorithm (Sentieon, Inc). The consensus sequence for each sample was generated from the alignment (BAM) and variant call format (VCF) files according to the following criteria: coverage from at least 5 unique reads with at least 80% of the reads supporting the call at the base position. For the bases that did not meet this criteria, an N was reported. A sequence is considered to pass quality criteria if it contains at most 30% N bases.

SARS-CoV-2 lineage designation

Viral sequences were assigned a PANGO lineage using pangolin (https://github.com/cov-lineages/pangolin). For this analysis, Pangolin data version v1.23.1 with Pangolin software v4.3.1 was used.

Supplementary Table 1. Health Systems Participating in the ViEW Network™.

Health system	**HealthPartners**	**Providence Health**	**Medical University of South Carolina**
Protocol number	0006-001	0006-001	0006-001
Institutional Review Board (IRB)	WCG IRB	WCG IRB	MUSC IRB
IRB approval number	20224919	20224919	Pro00129083
Geographic region	Minnesota and western Wisconsin	Eastern Washington and southern California	South Carolina
Sample size	5,466	883	202
Date range of patients included	September 24, 2023–January 20, 2024	September 24, 2023–January 21, 2024	September 24, 2023–January 13, 2024
Vaccine record data sources	Epic electronic health record, data linkage with the Minnesota Immunization Information Connection (MIIC), and medical and pharmacy claims data	Epic electronic health record and data linkages with the California Immunization Registry (CAIR) and Washington State Immunization Information System (WAIIS)	Epic electronic health record; immunization records in the South Carolina Statewide Immunization Online Network (SIMON) are reconciled in Epic after a patient's appointment
Informed consent	Waiver of consent obtained	Waiver of consent obtained	Waiver of consent obtained
Data privacy	Limited dataset stripped of direct identifiers	Limited dataset stripped of direct identifiers	Limited dataset stripped of direct identifiers

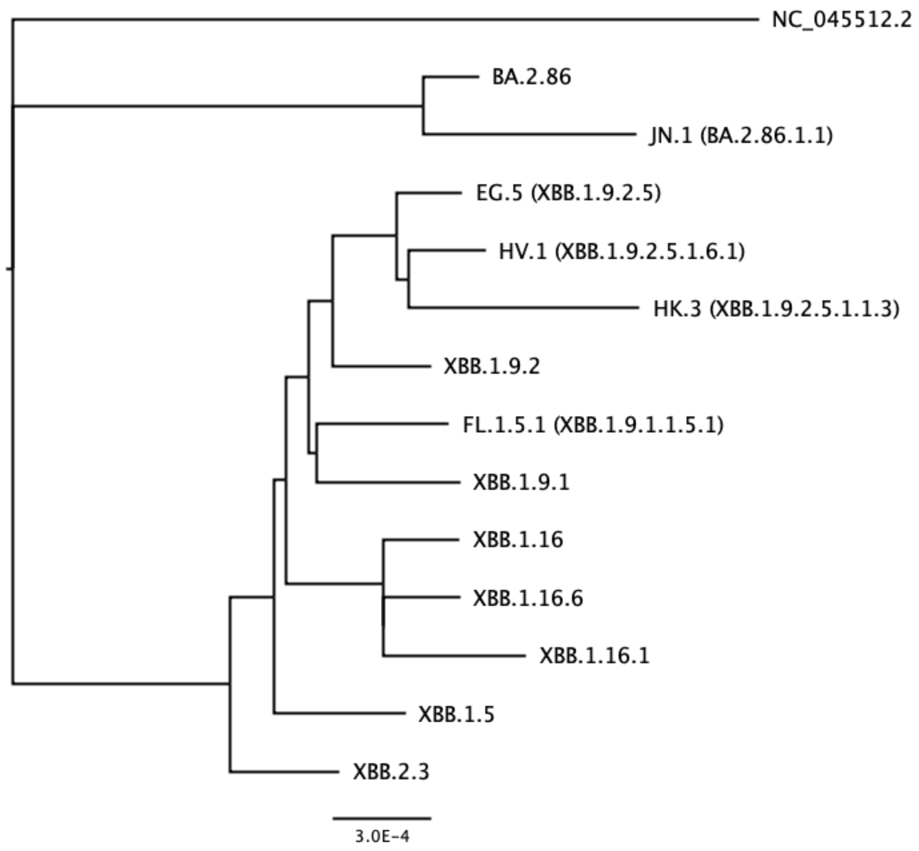

Supplementary Figure 1. Phylogram of SARS-CoV-2 lineage categories. The lengths of the branches are proportional to the genetic difference between variants. The distance bar shown represents 3 mutations per 10,000 bases. For each lineage, the most complete whole genome sequence from the earliest collection month associated with that lineage in our dataset was used. Sequence alignment and tree construction was carried out on the whole genome sequences using Augur, a tool developed by Nextstrain (https://docs.nextstrain.org/projects/augur/en/stable/). NC_045512.2 is the accession number for the originally published sequence for SARS-CoV-2, representing PANGO lineage “B”. Except for FL.1.5.1, sublineages of XBB.1.9.1 are aggregated with XBB.1.9.1. Except for HV.1 and HK.3, sublineages of EG.5 are aggregated with EG.5. Except for EG.5, HV.1, and HK.3, sublineages of XBB.1.9.2 are aggregated with XBB.1.9.2. Except for XBB.1.16.1 and XBB.1.16.6, sublineages of XBB.1.16 are aggregated with XBB.1.16. Except for JN.1, sublineages of BA.2.86 are aggregated with BA.2.86. Sublineages of each other named lineage are aggregated with the respective lineage.

SARS-CoV-2 variant	COVID-19 vaccination status	Inpatient and ED visits	Outpatient visits	OR (95% CI)	
		No. (%)	No. (%)	Unadjusted	Adjusted
Any	XBB.1.5 vaccine	96 (9.2)	310 (22.3)	—	—
	vs no XBB.1.5 vaccine	952 (90.8)	1,081 (77.7)	0.35 (0.28-0.45)	0.39 (0.29-0.52)
	vs BA.4/BA.5 vaccine	220 (21.0)	323 (23.2)	0.45 (0.34-0.61)	0.50 (0.36-0.70)
	vs wild-type vaccine	423 (40.4)	527 (37.9)	0.39 (0.30-0.50)	0.44 (0.32-0.60)
	vs unvaccinated	309 (29.5)	231 (16.6)	0.23 (0.17-0.31)	0.24 (0.17-0.34)
JN.1	XBB.1.5 vaccine	40 (23.0)	94 (39.8)	—	—
	vs no XBB.1.5 vaccine	134 (77.0)	142 (60.2)	0.45 (0.29-0.70)	0.52 (0.31-0.86)
	vs BA.4/BA.5 vaccine	33 (19.0)	58 (24.6)	0.75 (0.42-1.32)	0.84 (0.44-1.63)
	vs wild-type vaccine	45 (25.9)	54 (22.9)	0.51 (0.30-0.88)	0.68 (0.35-1.30)
	vs unvaccinated	56 (32.2)	30 (12.7)	0.23 (0.13-0.41)	0.20 (0.10-0.41)
XBB (any)	XBB.1.5 vaccine	28 (4.9)	117 (15.9)	—	—
	vs no XBB.1.5 vaccine	540 (95.1)	618 (84.1)	0.27 (0.18-0.42)	0.26 (0.16-0.42)
	vs BA.4/BA.5 vaccine	134 (23.6)	175 (23.8)	0.31 (0.20-0.50)	0.33 (0.20-0.56)
	vs wild-type vaccine	246 (43.3)	303 (41.2)	0.29 (0.19-0.46)	0.29 (0.17-0.48)
	vs unvaccinated	160 (28.2)	140 (19.0)	0.21 (0.13-0.34)	0.18 (0.10-0.30)
HV.1	XBB.1.5 vaccine	7 (5.0)	40 (20.6)	—	—
	vs no XBB.1.5 vaccine	134 (95.0)	154 (79.4)	0.20 (0.09-0.46)	0.14 (0.06-0.38)
	vs BA.4/BA.5 vaccine	38 (27.0)	49 (25.3)	0.23 (0.09-0.56)	0.18 (0.06-0.50)
	vs wild-type vaccine	55 (39.0)	62 (32.0)	0.20 (0.08-0.48)	0.13 (0.05-0.38)
	vs unvaccinated	41 (29.1)	43 (22.2)	0.18 (0.07-0.46)	0.13 (0.04-0.36)
EG.5	XBB.1.5 vaccine	8 (6.2)	21 (11.9)	—	—
	vs no XBB.1.5 vaccine	121 (93.8)	156 (88.1)	0.49 (0.21-1.15)	0.34 (0.12-0.96)
	vs BA.4/BA.5 vaccine	32 (24.8)	38 (21.5)	0.45 (0.18-1.16)	0.47 (0.15-1.45)
	vs wild-type vaccine	51 (39.5)	92 (52.0)	0.69 (0.28-1.66)	0.45 (0.15-1.35)
	vs unvaccinated	38 (29.5)	26 (14.7)	0.26 (0.10-0.68)	0.16 (0.05-0.52)

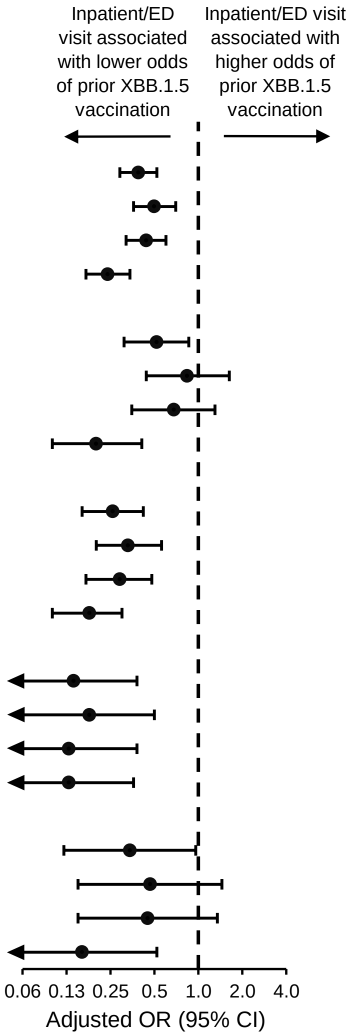

Supplementary Figure 2. Association between inpatient or emergency department vs outpatient visit type and prior receipt of an XBB.1.5 vaccine among older adults aged ≥65 years. Associations were calculated among all SARS-CoV-2 infections and among JN.1, XBB (any sublineage), HV.1, and EG.5 infections. Odds ratios (ORs) were calculated comparing prior receipt of an XBB.1.5 vaccine to no prior receipt of an XBB.1.5 vaccine (irrespective of previous COVID-19 vaccination history) as well as to each of three specific reference groups: 1) prior receipt of a BA.4/BA.5 vaccine but not an XBB.1.5 vaccine; 2) prior receipt of a wild-type vaccine but not a BA.4/BA.5 or XBB.1.5 vaccine; and 3) unvaccinated. Adjusted ORs were adjusted for age group (18-49, 50-64, 65-74, 75-84, and ≥85 years), sex, race and ethnicity (Asian, non-Hispanic; Black, non-Hispanic; Hispanic; white, non-Hispanic; and other/unknown), health system and state of residence, and collection date (natural cubic spline with 4 degrees of freedom). CI indicates confidence interval; ED, emergency department.

SARS-CoV-2 variant	COVID-19 vaccination status	Inpatient visits	Outpatient visits	OR (95% CI)	
		No. (%)	No. (%)	Unadjusted	Adjusted
Any	XBB.1.5 vaccine	73 (7.2)	558 (12.0)	—	—
	vs no XBB.1.5 vaccine	939 (92.8)	4,081 (88.0)	0.57 (0.44-0.73)	0.48 (0.36-0.66)
	vs BA.4/BA.5 vaccine	182 (18.0)	861 (18.6)	0.62 (0.46-0.83)	0.62 (0.44-0.89)
	vs wild-type vaccine	397 (39.2)	2,096 (45.2)	0.69 (0.53-0.90)	0.60 (0.43-0.84)
	vs unvaccinated	360 (35.6)	1,124 (24.2)	0.41 (0.31-0.54)	0.29 (0.21-0.41)
JN.1	XBB.1.5 vaccine	27 (19.4)	156 (20.0)	—	—
	vs no XBB.1.5 vaccine	112 (80.6)	624 (80.0)	0.96 (0.61-1.52)	0.71 (0.40-1.24)
	vs BA.4/BA.5 vaccine	26 (18.7)	158 (20.3)	1.05 (0.59-1.88)	0.89 (0.44-1.81)
	vs wild-type vaccine	34 (24.5)	281 (36.0)	1.43 (0.83-2.46)	1.47 (0.69-3.13)
	vs unvaccinated	52 (37.4)	185 (23.7)	0.62 (0.37-1.03)	0.31 (0.16-0.60)
XBB (any)	XBB.1.5 vaccine	23 (4.7)	187 (8.5)	—	—
	vs no XBB.1.5 vaccine	471 (95.3)	2,015 (91.5)	0.53 (0.34-0.82)	0.31 (0.19-0.53)
	vs BA.4/BA.5 vaccine	97 (19.6)	406 (18.4)	0.51 (0.32-0.84)	0.42 (0.24-0.75)
	vs wild-type vaccine	211 (42.7)	1,071 (48.6)	0.62 (0.40-0.99)	0.37 (0.21-0.64)
	vs unvaccinated	163 (33.0)	538 (24.4)	0.41 (0.25-0.65)	0.20 (0.11-0.34)
HV.1	XBB.1.5 vaccine	6 (5.2)	63 (11.0)	—	—
	vs no XBB.1.5 vaccine	110 (94.8)	510 (89.0)	0.44 (0.19-1.05)	0.25 (0.09-0.67)
	vs BA.4/BA.5 vaccine	25 (21.6)	128 (22.3)	0.49 (0.19-1.25)	0.37 (0.12-1.11)
	vs wild-type vaccine	47 (40.5)	247 (43.1)	0.50 (0.20-1.22)	0.22 (0.08-0.65)
	vs unvaccinated	38 (32.8)	135 (23.6)	0.34 (0.14-0.84)	0.18 (0.06-0.53)
EG.5	XBB.1.5 vaccine	7 (5.9)	34 (6.7)	—	—
	vs no XBB.1.5 vaccine	112 (94.1)	474 (93.3)	0.87 (0.38-2.02)	0.38 (0.13-1.11)
	vs BA.4/BA.5 vaccine	24 (20.2)	81 (15.9)	0.69 (0.27-1.77)	0.56 (0.17-1.83)
	vs wild-type vaccine	53 (44.5)	282 (55.5)	1.10 (0.46-2.60)	0.41 (0.13-1.29)
	vs unvaccinated	35 (29.4)	111 (21.9)	0.65 (0.27-1.60)	0.22 (0.07-0.72)

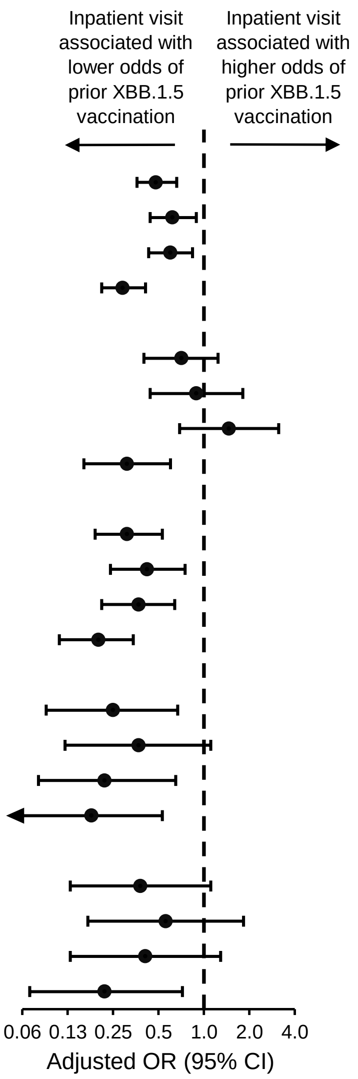

Supplementary Figure 3. Association between inpatient vs outpatient visit type and prior receipt of an XBB.1.5 vaccine. Associations were calculated among all SARS-CoV-2 infections and among JN.1, XBB (any sublineage), HV.1, and EG.5 infections. Odds ratios (ORs) were calculated comparing prior receipt of an XBB.1.5 vaccine to no prior receipt of an XBB.1.5 vaccine (irrespective of previous COVID-19 vaccination history) as well as to each of three specific reference groups: 1) prior receipt of a BA.4/BA.5 vaccine but not an XBB.1.5 vaccine; 2) prior receipt of a wild-type vaccine but not a BA.4/BA.5 or XBB.1.5 vaccine; and 3) unvaccinated. Adjusted ORs were adjusted for age group (18-49, 50-64, 65-74, 75-84, and ≥85 years), sex, race and ethnicity (Asian, non-Hispanic; Black, non-Hispanic; Hispanic; white, non-Hispanic; and other/unknown), health system and state of residence, and collection date (natural cubic spline with 4 degrees of freedom). CI indicates confidence interval.

SARS-CoV-2 variant	COVID-19 vaccination status	Inpatient and ED visits	Outpatient visits	OR (95% CI)	
		No. (%)	No. (%)	Unadjusted	Adjusted
Any	XBB.1.5 vaccine	113 (6.1)	538 (11.9)	—	—
	vs no XBB.1.5 vaccine	1,748 (93.9)	3,978 (88.1)	0.48 (0.39-0.59)	0.42 (0.32-0.54)
	vs BA.4/BA.5 vaccine	292 (15.7)	847 (18.8)	0.61 (0.48-0.78)	0.59 (0.45-0.79)
	vs wild-type vaccine	757 (40.7)	2,044 (45.3)	0.57 (0.46-0.71)	0.49 (0.38-0.64)
	vs unvaccinated	699 (37.6)	1,087 (24.1)	0.33 (0.26-0.41)	0.24 (0.18-0.32)
JN.1	XBB.1.5 vaccine	47 (15.7)	154 (20.2)	—	—
	vs no XBB.1.5 vaccine	253 (84.3)	608 (79.8)	0.73 (0.51-1.05)	0.62 (0.40-0.95)
	vs BA.4/BA.5 vaccine	45 (15.0)	156 (20.5)	1.06 (0.66-1.69)	0.98 (0.56-1.69)
	vs wild-type vaccine	97 (32.3)	273 (35.8)	0.86 (0.58-1.28)	0.80 (0.48-1.33)
	vs unvaccinated	111 (37.0)	179 (23.5)	0.49 (0.33-0.74)	0.31 (0.18-0.51)
XBB (any)	XBB.1.5 vaccine	35 (3.6)	185 (8.5)	—	—
	vs no XBB.1.5 vaccine	943 (96.4)	1,989 (91.5)	0.40 (0.28-0.58)	0.29 (0.19-0.44)
	vs BA.4/BA.5 vaccine	166 (17.0)	402 (18.5)	0.46 (0.31-0.69)	0.40 (0.25-0.64)
	vs wild-type vaccine	423 (43.3)	1,054 (48.5)	0.47 (0.32-0.69)	0.34 (0.22-0.53)
	vs unvaccinated	354 (36.2)	533 (24.5)	0.28 (0.19-0.42)	0.17 (0.11-0.27)
HV.1	XBB.1.5 vaccine	9 (3.9)	63 (11.2)	—	—
	vs no XBB.1.5 vaccine	219 (96.1)	498 (88.8)	0.32 (0.16-0.66)	0.22 (0.10-0.49)
	vs BA.4/BA.5 vaccine	46 (20.2)	127 (22.6)	0.39 (0.18-0.86)	0.34 (0.14-0.82)
	vs wild-type vaccine	96 (42.1)	238 (42.4)	0.35 (0.17-0.74)	0.22 (0.09-0.51)
	vs unvaccinated	77 (33.8)	133 (23.7)	0.25 (0.12-0.52)	0.15 (0.06-0.35)
EG.5	XBB.1.5 vaccine	8 (3.4)	34 (6.7)	—	—
	vs no XBB.1.5 vaccine	226 (96.6)	471 (93.3)	0.49 (0.22-1.08)	0.34 (0.14-0.83)
	vs BA.4/BA.5 vaccine	39 (16.7)	81 (16.0)	0.49 (0.21-1.15)	0.44 (0.17-1.18)
	vs wild-type vaccine	103 (44.0)	279 (55.2)	0.64 (0.29-1.42)	0.40 (0.16-1.02)
	vs unvaccinated	84 (35.9)	111 (22.0)	0.31 (0.14-0.71)	0.22 (0.08-0.56)

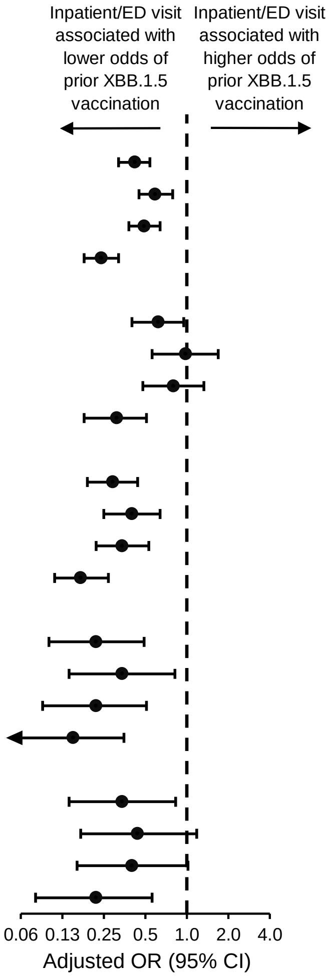

Supplementary Figure 4. Association between inpatient or emergency department vs outpatient visit type and prior receipt of an XBB.1.5 vaccine among SARS-CoV-2 mono-infected patients. Patients with a known respiratory virus coinfection were excluded (174), which included influenza (70), rhinovirus/enterovirus (42), RSV (28), human coronavirus (24), parainfluenza (9), adenovirus (3), human metapneumovirus (1), and bocavirus (1) coinfections. Associations were calculated among all SARS-CoV-2 infections and among JN.1, XBB (any sublineage), HV.1, and EG.5 infections. Odds ratios (ORs) were calculated comparing prior receipt of an XBB.1.5 vaccine to no prior receipt of an XBB.1.5 vaccine (irrespective of previous COVID-19 vaccination history) as well as to each of three specific reference groups: 1) prior receipt of a BA.4/BA.5 vaccine but not an XBB.1.5 vaccine; 2) prior receipt of a wild-type vaccine but not a BA.4/BA.5 or XBB.1.5 vaccine; and 3) unvaccinated. Adjusted ORs were adjusted for age group (18-49, 50-64, 65-74, 75-84, and ≥85 years), sex, race and ethnicity (Asian, non-Hispanic; Black, non-Hispanic; Hispanic; white, non-Hispanic; and other/unknown), health system and state of residence, and collection date (natural cubic spline with 4 degrees of freedom). CI indicates confidence interval; ED, emergency department.

SARS-CoV-2 variant	COVID-19 vaccination status	Inpatient and ED visits	Outpatient visits	OR (95% CI)	
		No. (%)	No. (%)	Unadjusted	Adjusted
Any	XBB.1.5 vaccine 7-59 days earlier	54 (2.8)	309 (6.7)	—	—
	vs no XBB.1.5 vaccine	1,795 (93.9)	4,081 (88.0)	0.40 (0.30-0.53)	0.35 (0.25-0.50)
	vs BA.4/BA.5 vaccine	294 (15.4)	861 (18.6)	0.51 (0.37-0.70)	0.51 (0.35-0.74)
	vs wild-type vaccine	783 (41.0)	2,096 (45.2)	0.47 (0.35-0.63)	0.41 (0.29-0.59)
	vs unvaccinated	718 (37.6)	1,124 (24.2)	0.27 (0.20-0.37)	0.21 (0.14-0.30)
	XBB.1.5 vaccine ≥60 days earlier	63 (3.3)	249 (5.4)	—	—
	vs no XBB.1.5 vaccine	1,795 (93.9)	4,081 (88.0)	0.58 (0.43-0.76)	0.49 (0.35-0.68)
	vs BA.4/BA.5 vaccine	294 (15.4)	861 (18.6)	0.74 (0.55-1.01)	0.71 (0.49-1.02)
	vs wild-type vaccine	783 (41.0)	2,096 (45.2)	0.68 (0.51-0.90)	0.58 (0.41-0.82)
	vs unvaccinated	718 (37.6)	1,124 (24.2)	0.40 (0.30-0.53)	0.29 (0.20-0.41)
JN.1	XBB.1.5 vaccine 7-59 days earlier	18 (5.9)	66 (8.5)	—	—
	vs no XBB.1.5 vaccine	257 (84.5)	624 (80.0)	0.66 (0.39-1.14)	0.65 (0.35-1.20)
	vs BA.4/BA.5 vaccine	46 (15.1)	158 (20.3)	0.94 (0.51-1.73)	1.01 (0.50-2.03)
	vs wild-type vaccine	98 (32.2)	281 (36.0)	0.78 (0.44-1.38)	0.85 (0.43-1.66)
	vs unvaccinated	113 (37.2)	185 (23.7)	0.45 (0.25-0.79)	0.32 (0.16-0.63)
	XBB.1.5 vaccine ≥60 days earlier	29 (9.5)	90 (11.5)	—	—
	vs no XBB.1.5 vaccine	257 (84.5)	624 (80.0)	0.78 (0.50-1.22)	0.60 (0.35-1.04)
	vs BA.4/BA.5 vaccine	46 (15.1)	158 (20.3)	1.11 (0.65-1.88)	0.95 (0.50-1.79)
	vs wild-type vaccine	98 (32.2)	281 (36.0)	0.92 (0.57-1.49)	0.80 (0.44-1.46)
	vs unvaccinated	113 (37.2)	185 (23.7)	0.53 (0.33-0.85)	0.30 (0.17-0.55)
XBB (any)	XBB.1.5 vaccine 7-59 days earlier	22 (2.2)	126 (5.7)	—	—
	vs no XBB.1.5 vaccine	956 (96.5)	2,015 (91.5)	0.37 (0.23-0.58)	0.28 (0.17-0.47)
	vs BA.4/BA.5 vaccine	166 (16.8)	406 (18.4)	0.43 (0.26-0.70)	0.39 (0.23-0.68)
	vs wild-type vaccine	429 (43.3)	1,071 (48.6)	0.44 (0.27-0.70)	0.33 (0.19-0.56)
	vs unvaccinated	361 (36.4)	538 (24.4)	0.26 (0.16-0.42)	0.17 (0.10-0.29)
	XBB.1.5 vaccine ≥60 days earlier	13 (1.3)	61 (2.8)	—	—
	vs no XBB.1.5 vaccine	956 (96.5)	2,015 (91.5)	0.45 (0.25-0.82)	0.28 (0.14-0.55)
	vs BA.4/BA.5 vaccine	166 (16.8)	406 (18.4)	0.52 (0.28-0.97)	0.40 (0.19-0.81)
	vs wild-type vaccine	429 (43.3)	1,071 (48.6)	0.53 (0.29-0.98)	0.33 (0.16-0.67)
	vs unvaccinated	361 (36.4)	538 (24.4)	0.32 (0.17-0.59)	0.17 (0.08-0.34)

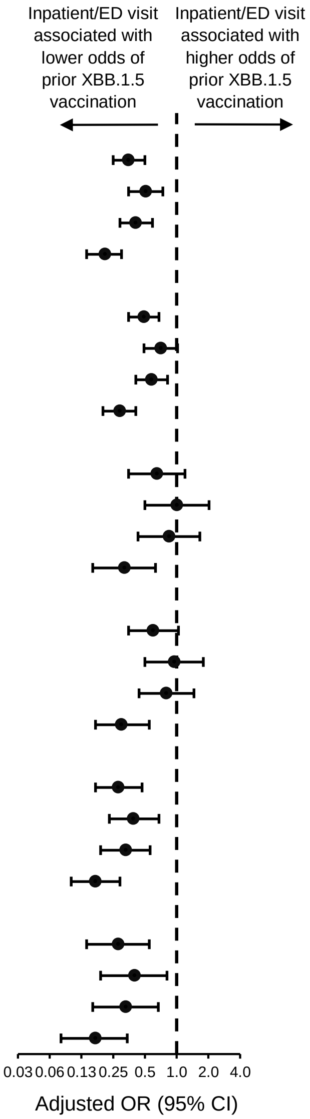

Supplementary Figure 5. Association between inpatient or emergency department vs outpatient visit type and prior receipt of an XBB.1.5 vaccine 7-59 and ≥60 days earlier. Associations were calculated among all SARS-CoV-2 infections and among JN.1 and XBB (any sublineage) infections. Odds ratios (ORs) were calculated comparing both receipt of an XBB.1.5 vaccine 7-59 days earlier and receipt of an XBB.1.5 vaccine ≥60 days earlier to no prior receipt of an XBB.1.5 vaccine (irrespective of previous COVID-19 vaccination history) as well as to each of three specific reference groups: 1) prior receipt of a BA.4/BA.5 vaccine but not an XBB.1.5 vaccine; 2) prior receipt of a wild-type vaccine but not a BA.4/BA.5 or XBB.1.5 vaccine; and 3) unvaccinated. Adjusted ORs were adjusted for age group (18-49, 50-64, 65-74, 75-84, and ≥85 years), sex, race and ethnicity (Asian, non-Hispanic; Black, non-Hispanic; Hispanic; white, non-Hispanic; and other/unknown), health system and state of residence, and collection date (natural cubic spline with 4 degrees of freedom). CI indicates confidence interval; ED, emergency department.

SARS-CoV-2 variant	COVID-19 vaccination status	Inpatient and ED visits	Outpatient visits	OR (95% CI)	
		No. (%)	No. (%)	Unadjusted	Adjusted
Any	Unvaccinated	718 (37.6)	1,124 (24.2)	1.00 [Reference]	1.00 [Reference]
	Wild-type vaccine	783 (41.0)	2,096 (45.2)	0.58 (0.52-0.66)	0.50 (0.43-0.59)
	BA.4/BA.5 vaccine	294 (15.4)	861 (18.6)	0.53 (0.45-0.63)	0.41 (0.33-0.50)
	XBB.1.5 vaccine	117 (6.1)	558 (12.0)	0.33 (0.26-0.41)	0.24 (0.19-0.32)
JN.1	Unvaccinated	113 (37.2)	185 (23.7)	1.00 [Reference]	1.00 [Reference]
	Wild-type vaccine	98 (32.2)	281 (36.0)	0.57 (0.41-0.79)	0.38 (0.25-0.58)
	BA.4/BA.5 vaccine	46 (15.1)	158 (20.3)	0.48 (0.32-0.71)	0.32 (0.19-0.53)
	XBB.1.5 vaccine	47 (15.5)	156 (20.0)	0.49 (0.33-0.74)	0.31 (0.19-0.52)
XBB (any)	Unvaccinated	361 (36.4)	538 (24.4)	1.00 [Reference]	1.00 [Reference]
	Wild-type vaccine	429 (43.3)	1,071 (48.6)	0.60 (0.50-0.71)	0.51 (0.41-0.63)
	BA.4/BA.5 vaccine	166 (16.8)	406 (18.4)	0.61 (0.49-0.76)	0.42 (0.32-0.56)
	XBB.1.5 vaccine	35 (3.5)	187 (8.5)	0.28 (0.19-0.41)	0.17 (0.11-0.26)
HV.1	Unvaccinated	78 (33.9)	135 (23.6)	1.00 [Reference]	1.00 [Reference]
	Wild-type vaccine	97 (42.2)	247 (43.1)	0.68 (0.47-0.98)	0.66 (0.42-1.04)
	BA.4/BA.5 vaccine	46 (20.0)	128 (22.3)	0.62 (0.40-0.96)	0.43 (0.24-0.74)
	XBB.1.5 vaccine	9 (3.9)	63 (11.0)	0.25 (0.12-0.52)	0.15 (0.06-0.34)
EG.5	Unvaccinated	85 (35.7)	111 (21.9)	1.00 [Reference]	1.00 [Reference]
	Wild-type vaccine	106 (44.5)	282 (55.5)	0.49 (0.34-0.70)	0.56 (0.35-0.88)
	BA.4/BA.5 vaccine	39 (16.4)	81 (15.9)	0.63 (0.39-1.01)	0.49 (0.27-0.91)
	XBB.1.5 vaccine	8 (3.4)	34 (6.7)	0.31 (0.14-0.70)	0.22 (0.08-0.56)

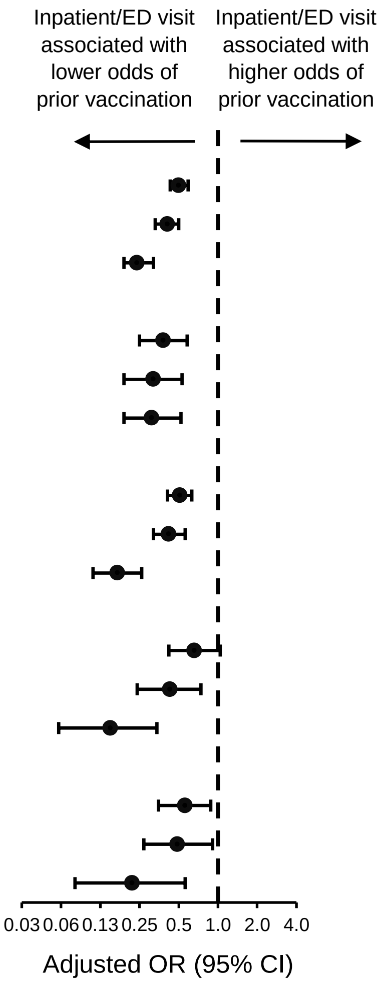

Supplementary Figure 6. Association between inpatient or emergency department vs outpatient visit type and prior receipt of a wild-type, BA.4/BA.5, or XBB.1.5 vaccine, each vs unvaccinated. Associations were calculated among all SARS-CoV-2 infections and among JN.1, XBB (any sublineage), HV.1, and EG.5 infections. Odds ratios (ORs) were calculated comparing each of three groups to unvaccinated: 1) prior receipt of a wild-type vaccine but not a BA.4/BA.5 or XBB.1.5 vaccine; 2) prior receipt of a BA.4/BA.5 vaccine but not an XBB.1.5 vaccine; and 3) prior receipt of an XBB.1.5 vaccine. Adjusted ORs were adjusted for age group (18-49, 50-64, 65-74, 75-84, and ≥85 years), sex, race and ethnicity (Asian, non-Hispanic; Black, non-Hispanic; Hispanic; white, non-Hispanic; and other/unknown), health system and state of residence, and collection date (natural cubic spline with 4 degrees of freedom). CI indicates confidence interval; ED, emergency department.
